# Molecular Biosensing Mechanisms in the Spleen for the Removal of Aged and Damaged Red Cells from the Blood Circulation

**DOI:** 10.3390/s100807099

**Published:** 2010-07-27

**Authors:** Yoshiaki Sugawara, Yuko Hayashi, Yuki Shigemasa, Yoko Abe, Ikumi Ohgushi, Eriko Ueno, Fumio Shimamoto

**Affiliations:** Department of Health Science, Prefectural University of Hiroshima, Hiroshima 734-8558, Japan; E-Mails: chil05k.zutto--saranheyo-yuko@docomo.ne.jp (Y.H.); commonplace32@yahoo.co.jp (Y.S); onitsukaline.14@ezweb.ne.jp (Y.A.); nirenum.hy.rhcp-255922@ezweb.ne.jp (I.O.); eriko.u-everblue@cup.ocn.ne.jp (E.U.); simamoto@pu-hiroshima.ac.jp (F.S.)

**Keywords:** biosensing, Heinz body formation, hemichrome formation, normal human erythrocytes, spleen

## Abstract

Heinz bodies are intraerythrocytic inclusions of hemichrome formed as a result of hemoglobin (Hb) oxidation. They typically develop in aged red cells. Based on the hypothesis that hemichrome formation is an innate characteristic of physiologically normal Hb molecules, we present an overview of our previous findings regarding the molecular instability of Hb and the formation of hemichrome, as well as recent findings on Heinz body formation within normal human erythrocytes. Human adult Hb (HbO_2_ A) prepared from healthy donors showed a tendency to produce hemichrome, even at close to physiological temperature and pH. Recent studies found that the number of Heinz bodies formed in red cells increased with increasing temperature when freshly drawn venous blood from healthy donors was subjected to mild heating above 37 °C. These findings suggest that Hb molecules control the removal of non-functional erythrocytes from the circulation via hemichrome formation and subsequent Heinz body clustering. In this review, we discuss the molecular biosensing mechanisms in the spleen, where hemichrome formation and subsequent Heinz body clustering within erythrocytes play a key role in the removal of aged and damaged red cells from the blood circulation.

## 1. Introduction

Human red blood corpuscles survive in the circulation for an average of 120 days. Removal of aged and damaged red cells from the blood circulation is essential for its homeostasis. Heinz bodies are intraerythrocytic inclusions of hemichrome formed from oxidized or denatured hemoglobin (Hb), and are typically formed in aged red cells. However, they have also been found and characterized in drug-induced hemolytic anemia, defects in the intraerythrocytic reducing system (e.g., glucose 6-phosphate dehydrogenase [G-6-PD] deficiency) and in unstable Hb disease [1,2]. Heinz bodies have rarely been mentioned in the context of normal Hb or normal erythrocytes. However, Heinz bodies in normal erythrocytes are of interest, because they, or their related intraerythrocytic inclusions, are involved in the recognition mechanisms in the spleen responsible for the removal of non-functional erythrocytes from the circulation. The rigid intraerythrocytic hemichrome inclusions are known to act as “sticking points”, and hence Heinz body-containing red cells become trapped and undergo hemolysis [3].

Hemichrome is rarely found in erythrocytes *in situ*, even though the reaction dynamics of Hb with molecular oxygen (O_2_) make it a particularly suitable O_2_ carrier. Hb can bind O_2_ in ferrous form to carry out its physiological functions. During this reversible O_2_ binding, the oxygenated form of Hb (HbO_2_) is known to be oxidized by the bound oxygen to the ferric met form (metHb), which cannot be oxygenated, and is thus physiologically inactive. Although metHb is reduced back to the ferrous state by an intraerythrocytic nicotinamide adenine dinucleotide (NADH)-dependent reducing system, it has been suggested that its oxidation (autoxidation) process can be followed by transformation of the oxidized molecule (high-spin Fe^3+^) into a species absorbing as a low-spin compound, *i.e*., hemichrome, the formation of which can result in the accumulation of soluble and insoluble hemichromes and precipitation [4–9]. Despite these findings, direct evidence of hemichrome formation in normal erythrocytes is lacking.

Hemichrome formation is enhanced in separated α and β chains, compared with the tetrameric parent Hb [5,9–12]. Following the method of Brunori *et al.* [10], our previous spectroscopic study [13] showed that human adult Hb (HbO_2_ A) from healthy donors tended to degrade to produce hemichrome, even at close to physiological temperatures and pH. However, its occurrence was a function of pH, temperature and progress of autoxidation of ferrous HbO_2_ A to the ferric met form, through oxidation by bound oxygen.

Based on the hypothesis that the instability of oxidized Hb that leads to hemichrome formation is not only a peculiarity of labile Hb in patients, but is also an innate characteristic of physiologically normal Hb molecules, the present review examines our previous findings on the molecular instability of Hb and its degradation to hemichrome, as well as the recent findings on Heinz body formation within normal human erythrocytes. The latter study [14] investigated the possibility of Heinz body formation occurring during mild heating of blood samples *in vitro*, at close to physiological temperatures. The changes in normal human red blood corpuscles during mild heating were examined by light microscopy under oil-immersion. The number of Heinz bodies formed in red cells increased with increasing temperature. These observations, combined with the results of our previous study, suggest that Hb molecules control the removal of non-functional erythrocytes from the circulation through hemichrome formation and subsequent Heinz body clustering. In this overview, we examine the molecular biosensing mechanisms in the spleen responsible for the removal of aged and damaged red cells from the blood circulation.

## 2. Innate Instability of Hb Molecule and Degradation to Hemichrome

In this section, we discuss the results of our previous spectrophotometric study of hemichrome formation from human HbO_2_ A, in 0.1 M buffer at various temperatures and pH values [13]. HbO_2_ A was prepared from freshly drawn human blood samples (total 50–80 mL) obtained from healthy donors. Samples were centrifuged at 2,400 g for 10 min to remove supernatant plasma and buffy coats. The erythrocytes obtained were washed five times with ice-cooled 0.9% NaCl solution (saline) by centrifugation and hemolysed by adding the same volume of ice-cooled distilled water. The hemolysate was then fractionated with ammonium sulfate between 20% and 70% saturation at pH 6.8. After dialysis, this solution was passed through two Sephadex G-50 columns (5 × 90 cm) equilibrated with 10 mM Tris-HCl (pH 8.6). The effluent Hb fraction was further separated using a diethylaminoethyl-cellulose column (3 × 15 cm) equilibrated with 10 mM Tris-HCl (pH 8.6) and washed sequentially with stepwisely changing the buffer solutions as follows: (1) 60 mM Tris-HCl (pH 8.0); (2) 100 mM Tris-HCl (pH 7.5); finally (3) 100 mM NaCl with 100 mM Tris-HCl (pH 8.0). HbO_2_ A was eluted out as the major fraction with 60 mM Tris-HCl (pH 8.0) and used in the experiments after dialyzing against 5 mM Tris-HCl buffer (pH 8.5).

Hemichrome formation was observed spectroscopically in 0.1 M buffer over wide pH (4.5–10.5) and temperature ranges (35–55°C). Two milliliters of solution containing 0.2 M buffer was placed in a test tube and incubated in a water bath maintained at each desired temperature (± 0.1°C) using a NESLAB temperature control (Model RTE-100 or 111 or 210; NESLAB Instruments, Inc., Portsmouth, NH, USA). The reaction was started by adding the same volume of fresh HbO_2_ A solution (125–235 μM in heme contents). For spectrophotometry, the reaction mixture was then quickly transferred to a spectrophotometric cell (Spectrocell, Type Inject-A-Cell; Funakoshi Co., Tokyo, Japan) with a screw-cap-stopper, and changes in absorption at 450–650 nm were recorded on the same chart at measured time intervals. Spectra were recorded using a UV/VIS spectrophotometer (JASCO, Model Ubest-50 or V-560 or V-570; Japan Spectroscopic Co., Tokyo, Japan), equipped with a thermostatically controlled (within ± 0.1 °C) cell holder. At the final state of each run, Hb molecules were all completely converted to the ferric met form by the addition of potassium ferricyanide. The buffers used were: acetate for pH 4.5–5.5, 2-(*N*-morpholino) ethanesulfonic acid monohydrate (MES) for pH 5.0–6.75, *N*-2-hydroxyethylpiperazine-*N*′-2-ethanesulfonic acid for pH 6.55–8.3, 2-(cyclohexylamino) ethanesulfonic acid for pH 8.2–10.2, and 3-cyclohexylaminopropanesulfonic acid for pH 10.0–10.5.

**Figure 1.**
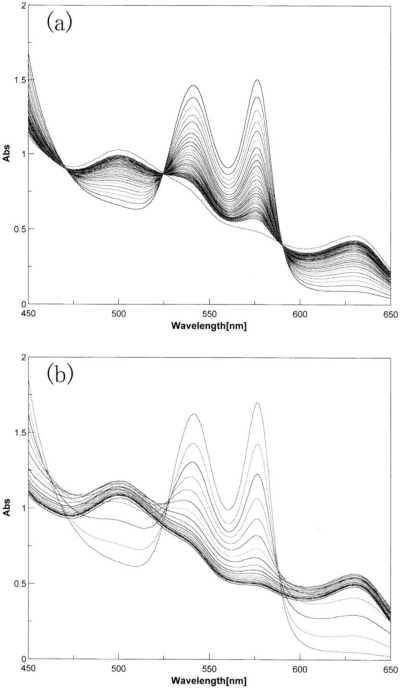
Spectral changes over time associated with hemichrome formation for human HbO_2_ A redrawn from Sugawara *et al.* [13]. **(a)** Monitoring in 0.1 M MES buffer (pH 5.0) at 37 °C and **(b)** in 0.1 M MES buffer (pH 6.5) at 40 °C. In case a, hemichrome formation was not detected until the reaction was almost 75% complete. In case b, the emergence of hemichrome due to accumulation and precipitation of soluble and insoluble hemichromes was noticeable by sudden disruption of the recorded spectrum at the final stage (at around 17.5 h) of the time course. Conditions: HbO_2_ concentration was 235 μM (in heme contents); and scanning intervals were (a) 15 min and (b) 150 min.

Figure 1 shows some examples of spectrophotometric changes in hemichrome formation during autoxidation of HbO_2_ A; its occurrence is inseparably related to the autoxidation process. HbO_2_ A can be oxidized to its ferric met form through the tendency of the bound dioxygen to oxidize ferrous heme iron (II), with generation of superoxide anions, as shown by the following reaction:

$${\text{Hb(II)O}}_{2}\overset{k_{A}}{\to }\text{metHb(III) }+\;{4(O}_{2}{}^{-}\text{)}$$

where *k*_A_ represents the observed rate constant of the autoxidation at a given pH and temperature. As shown in Figure 1a, the observed spectra proceeded with time with no evidence of hemichrome formation during the entire process when fresh HbO_2_ A was placed in 0.1 M MES buffer (pH 5.0) at 37 °C. However, when HbO_2_ was incubated in 0.1 M MES buffer (pH 6.5) at 40 °C, the situation was very different (Figure 1b). During the late stage of autoxidation, a sudden disruption of the recorded spectra was observed; autoxidation was occurring, but hemichrome formation could be detected by an elevation of the base line and a shift of the isosbestic points, caused by precipitation.

**Figure 2.**
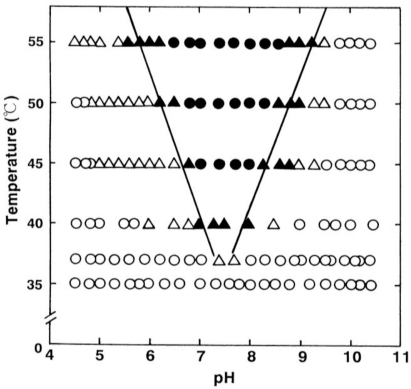
pH-temperature diagram of hemichrome formation for human HbO_2_ A, redrawn from Sugawara *et al.* [13]. The symbols represent: •- hemichrome formation noticeable at initial stage during the course of autoxidation; ▴- at intermediate stage; Δ- at final stage; ○- autoxidation reaction proceeded with no hemichrome formation during the entire process.

Hemichrome formation during autoxidation was examined spectrophotometrically while varying the temperature of the solution from 35 °C to 55 °C and the pH from 4.5 to 10.5. Hemichrome formation could be observed at every stage during the course of autoxidation, *i.e.*, during the initial, intermediate, and final stages, as a function of pH and temperature of the solution. A diagram illustrating the phenomenon is depicted in Figure 2.

The phenomenon was not simple, and its occurrence was a function of not only pH and temperature of the solution, but also of the progress of autoxidation of HbO_2_ A. We therefore attempted to categorize the phenomenon into the following four cases in terms of [HbO_2_]*_t= E.P._* /[HbO_2_]_0_:

1. *t* = 0 or [HbO_2_]*_t= E.P._* /[HbO_2_]_0_ = 1 ≤ t_E.P._ < [HbO_2_]*_t= E.P._* /[HbO_2_]_0_ = 0.75;
2. [HbO_2_]*_t= E.P._* /[HbO_2_]_0_ = 0.75 ≤ t_E.P_ < [HbO_2_]*_t= E.P._* /[HbO_2_]_0_ = 0.25;
3. t_E.P._ ≤ [HbO_2_]*_t= E.P._* /[HbO_2_]_0_ = 0.25;
4. no hemichrome formation during the entire process.

*E.P.* is the observed emergence point of hemichrome formation in each run. [HbO_2_]*_t= E.P._* /[HbO_2_]_0_ is the ratio of HbO_2_ concentration after time *t = E.P.* to that at time *t* = 0 and can be monitored by the absorbance ratio of (A*_t_* − A_∞_)/(A_0_ − A_∞_) at 576 nm (α-peak of HbO_2_ A). Since [HbO_2_]*_t= E.P._*/[HbO_2_]_0_ = 0.5 represents equal mixtures of HbO_2_ and metHb, *i.e.*, the midpoint of the autoxidation reaction, case 1 means that hemichrome formation was noticeable at the initial stage of autoxidation. Accordingly, case 2 indicates its occurrence at the intermediate stage, and case 3 at the final stage. In Figure 2, the symbols used correspond to: • for case 1, ▴ for case 2, Δ for case 3 and ○ for case 4, respectively. To determine if the phenomenon was represented by case 3 or case 4, the reaction mixture was converted to metHb by the addition of small amounts of ferricyanide and maintained at the given temperature for 2 days to see whether hemichrome precipitation occurred.

Thus, the findings shown in Figure 2 suggest that HbO_2_ A was highly susceptible to hemichrome formation, even under physiological temperature and pH. In Figure 2, solid lines show the threshold for this susceptibility in relation to pH and temperature. When compared with the tetrameric parent Hb molecules, the isolated α and β chains were found to have much higher susceptibilities to hemichrome formation, and showed individual pH-temperature diagrams [13]. Even though hemichrome formation is a function of pH, temperature, and progress of autoxidation, the phenomenon can be described in air-saturated conditions as:

$${\text{Hb(II)O}}_{2}\overset{k_{A}}{\to }\text{metHb(III)}+4(O_{2}{}^{-})\to \text{hemichrome}$$

As described elsewhere [13,15], electron paramagnetic resonance measurements were carried out for the resulting oxidation products of isolated β chains over a magnetic field of 0–500 mT at 8.0 K in 10 mM maleate buffer (pH 6.2) and in the presence of 50% (v/v) glycerol. They demonstrated a low-spin spectrum with *g* values of *g*_1_ = 2.77, *g*_2_ = 2.27, and *g*_3_ = 1.68, in addition to the usual aquo-met species with *g* values of 5.86 and 1.99. According to Rifkind *et al.* [9], such low-spin complexes characterized by the highest *g* values in the range of 2.83−2.75 and the lowest *g* values in the range of 1.69−1.63 have been designated as complex B, indicating the crystal field parameters of the reversible hemichrome, namely a water-retained bis-histidine complex. The molar fraction of the hemichrome (complex B) in the oxidized β chains was estimated to be 85% at pH 6.2, as a low-spin species was in equilibrium with a high-spin species corresponding to the usual aquo-met species.

## 3. Innate Instability of Hb Molecule, its Degradation to Hemichrome, and Subsequent Heinz Body Formation in Normal Human Erythrocytes during Mild Heating

In this section, we discuss the results of our study investigating Heinz body formation in normal human erythrocytes [14]. Aliquots of freshly drawn venous blood from healthy donors were subjected to mild heating at temperatures above 37 °C for 30 min, to investigate hemichrome formation and subsequent Heinz body formation in normal human erythrocytes. Heinz bodies were visualized by exposing blood smears to acetylphenylhydrazine and stained with crystal violet. Changes within the erythrocytes were observed using light microscopy under oil-immersion. Prior to the Heinz body formation test (acetylphenylhydrazine test) [16,17], the blood samples were subjected to mild heating *in vitro*. A 2-mL sample was placed in a test tube and incubated in a water bath maintained at each desired temperature (±0.1 °C) above 37 °C for 30 min, using NESLAB temperature control (Model RTE-100 or 111 or 210 or 221).

β-Acetylphenylhydrazine and crystal violet (research grade for pathology) were used (Wako Pure Chemical, Co., Osaka, Japan) for the procedure, as described by Bauer [17]. A 0.1-mL aliquot of the blood sample was suspended in 2 mL of acetylphenylhydrazine solution, in which 100 mg of acetylphenylhydrazine and 200 mg of glucose in 100 mL of 0.067 M phosphate buffer were dissolved at pH 7.6. Using a “blowout” pipette, the solution was aerated two or three times by drawing it up into the pipette and blowing it out, together with a small quantity of air. This mixture was incubated at 37 °C for 2 h. Aeration was repeated halfway through and immediately after the 2 h incubation. A drop (approximately 10 μL) of the resulting mixture was placed on a cover glass, which was then inverted onto a microscope slide containing 30 μL of crystal violet solution. The smear was allowed to stand for 20 min in wet preparation at room temperature and subsequently examined under a light microscope (Nikon Model-FXA; Nikon Co., Tokyo, Japan) equipped with a digital camera (Olympus Model-DP-70; Olympus Co., Tokyo, Japan). Photomicrographs were taken under oil-immersion at 1,000 × magnification. Subsequently, the number of Heinz bodies per cell was counted in 100 red cells per view.

**Figure 3.**
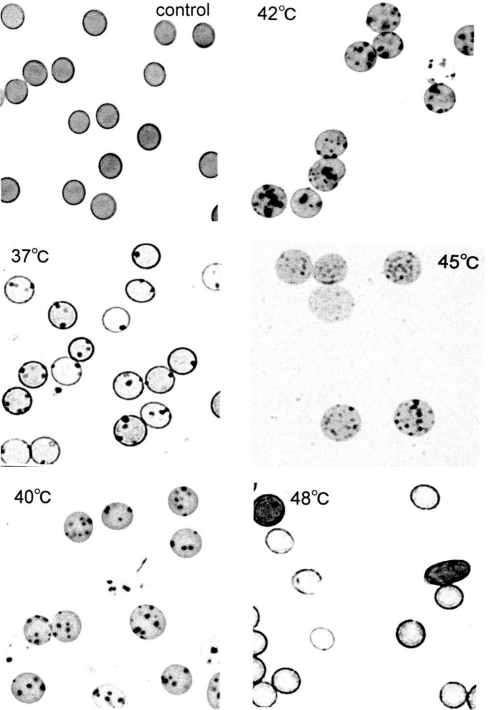
Microscopic views of Heinz bodies after mild heating of blood samples obtained from one healthy donor, redrawn from Sugawara *et al.* [14]. Using a temperature-untreated sample as a control, aliquots of freshly drawn venous blood were subjected to mild heating at 37 °C, 40 °C, 42 °C, 45 °C or 48 °C for 30 min. Heinz bodies were then visualized by exposure to acetylphenylhydrazine and dyeing with crystal violet. The changes that occurred within erythrocytes were observed by light microscopy under oil-immersion.

**Figure 4.**
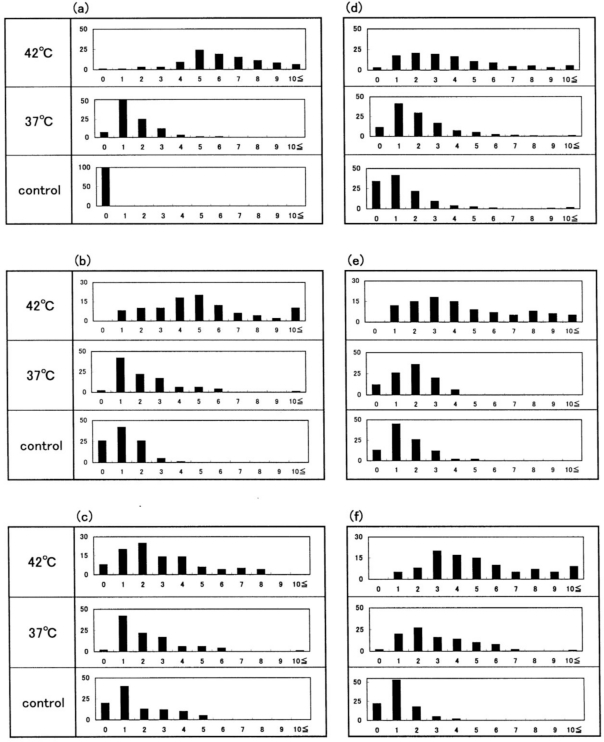
Histograms represent Heinz bodies detected in blood samples after mild heating above 37 °C, redrawn from Sugawara *et al.* [14]. The histograms for 37 °C and 42 °C are displayed using a temperature-untreated sample as a reference. One hundred red cells were chosen and the number of Heinz bodies contained in each red cell was counted. Figure 4a was constructed from the microscopic views shown in Figure 3.

Figure 3 shows representative images of Heinz bodies within normal red corpuscles obtained from a healthy donor during mild heating to a given temperature. The blood sample used as a temperature-untreated reference was kept at a low temperature (0–4 °C) for as long as possible after withdrawal, and never exposed to temperatures above room temperature. Heinz bodies were observed in each smear. Some of the changes are shown in Figure 4a; histograms of samples treated at 37 °C and 42 °C are displayed with a temperature-untreated sample as a reference, based on the number of Heinz bodies contained in each of 100 red cells per view. These results confirmed that Heinz body formation increased with increasing temperature of the blood samples over 37 °C. Red cells in blood samples exposed to 48 °C were hemolyzed.

**Figure 5.**
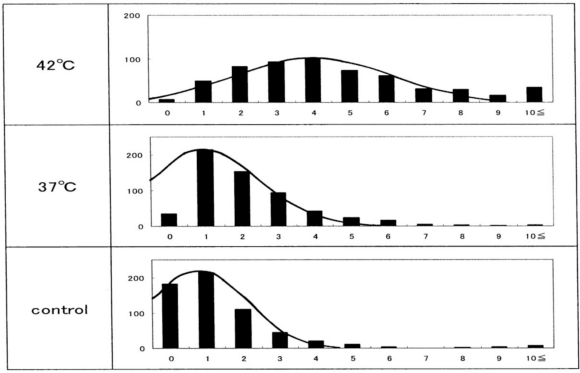
Histograms obtained from the arithmetic summation of all histograms shown in Figure 4, redrawn from Sugawara *et al.* [14]. The solid lines show the computed curve obtained by the least-squares method using a Gaussian curve. The resulting Gaussian constants are summarized in the text.

The other histograms shown in Figure 4 represent the results from another five healthy donors, with a temperature-untreated sample as a reference. Although there was considerable inter-individual variation, temperature-dependent Heinz body formation was apparent in all preparations. Arithmetic summation was used for all the histograms to reduce the variability and allow a better assessment of temperature-dependent Heinz body formation. The results of this summation are shown in Figure 5. Solid lines represent the curves derived using the least-squares method using a Gaussian curve. Each computed curve showed satisfactory-to-good agreement with the experimental data over the entire range. The following is a summary of the Gaussian constants resulting from the least-squares fitting: a (peak height) = 101, b (peak width at half of maximum) = 5.0 and c (peak location) = 4.0 for blood samples treated at 42 °C; a = 215, b = 3.5 and c = 1.0 for blood samples treated at 37°C; and a = 215, b = 3.0 and c = 0.8 for temperature-untreated blood samples.

## 4. *In vitro* Evaluation of Blood Fluidity during Mild Heating Using a Micro-channel Array Flow Analyzer to Give an Index of Erythrocyte Deformability

**Figure 6.**
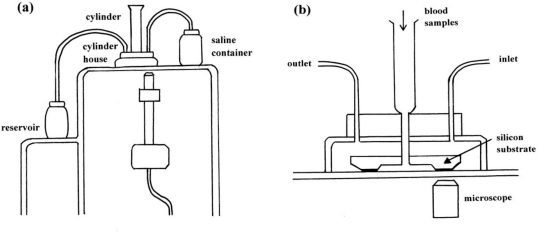
External appearance **(a)** of a micro-channel array flow analyzer (MC-FAN) and diagram **(b)** of cylinder house. Blood samples were allowed to flow through the cylinder house by applying a pressure difference of 20 cm H_2_O (*i.e.*, the difference between saline container and reservoir).

Kikuchi *et al.* described a tool for measuring blood fluidity or blood rheology [18] in 1992, and this has subsequently been implemented in numerous studies [19–26]. Figure 6 shows a diagram of this tool, called a micro-channel array flow analyzer (MC-FAN). It includes a characteristic V-shaped groove array in an integral circuit, with 8,736 flow paths (width, 7 μm; length, 30 μm; depth, 4.5 μm) engraved on a 15 × 15 × 0.5-mm single-crystal silicon substrate, using an anisotropic etching technique. The array is housed in a cylinder. This equipment makes it possible to not only observe red cells passing through individual micro-channel arrays by use of an inverted metallographic microscope, but also to evaluate blood fluidity through the groove array in terms of transit time of the blood sample for a given transit sample volume. Flow rate can be determined by timing when the blood sample meniscus crosses graduation marks at 10 μL intervals from 0–100 μL. Transit sample volume was therefore equivalent to loss of the blood sample within the cylinder. Here we review our *in vitro* findings on blood fluidity during mild heating, using a MC-FAN (type-HR300; Hitachi Haramachi Electronics Co., Ibaraki, Japan).

Donors were male or female student volunteers from the Prefectural University of Hiroshima, aged 18–22 years. All volunteers were aware of the aims and procedures of the study and gave their informed consent to participate, as approved by our Institutional Ethical Review Board. Nine aliquots of freshly drawn venous blood samples (10 mL in total) were obtained from healthy donors and mixed with one aliquot of 3.2% sodium citrate (Na_3_C_6_H_5_O_7_ 2H_2_O) or 3.8% sodium citrate (Na_3_C_6_H_5_O_7_ 5H_2_O). Blood samples were centrifuged at 400 *g* for 10 min at 0–4 °C, to eliminate the possibility of interference with blood fluidity measurements caused by platelet aggregation onto micro-channel flow paths. The supernatant (platelet-rich plasma, PRP) was then discarded using a narrow Teflon-lined capillary connected to a water-jet pump. The remainder (PRP-removal blood) was subjected to mild heating, prior to application to the MC-FAN. A 2-ml aliquot was placed in a test tube and incubated in a water bath maintained at each desired temperature (± 0.1 °C) above 37 °C for 30 min, using NESLAB temperature control (Model RTE-100 or 111 or 210 or 221).

**Figure 7.**
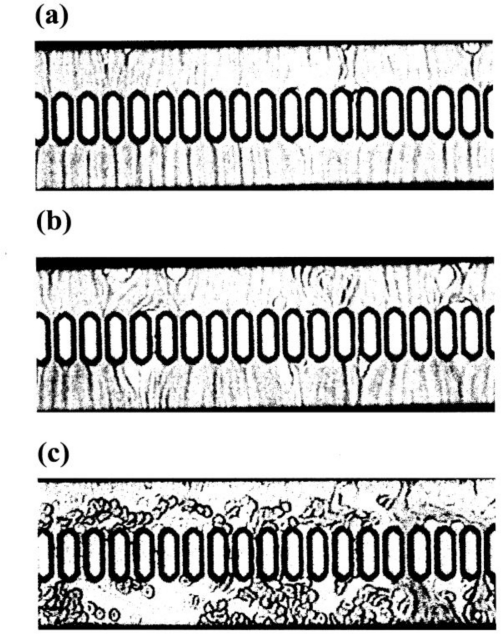
Microscopic details of blood samples passing through individual micro-channel array of the MC-FAN. **(a)** View of temperature-untreated sample with transit time = 12.6 s and transit sample volume = 25 μL. **(b)** Identical temperature-untreated with transit time = 27.8 s and transit sample volume = 50 μL. **(c)** View of identical blood sample to that in a and b, but subjected to mild heating at 45 °C for 30 min prior to application to MC-FAN, with a transit time = 120.7 s and transit sample volume = 30 μL.

**Figure 8.**
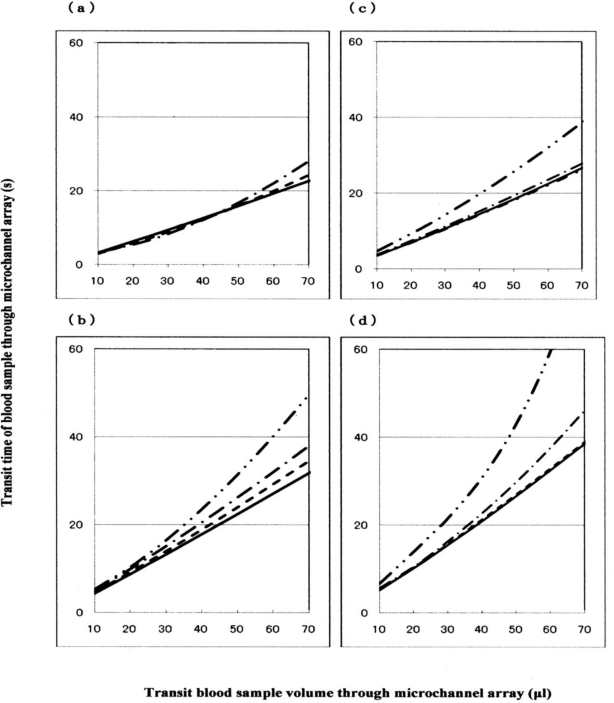
Representative examples (*n* = 4) of blood fluidity measured in terms of transit time against transit sample volume. Solid lines represent the temperature-untreated control; broken lines represent samples treated at 37 °C; one-point lines represent samples treated at 42 °C; and two-point lines represent samples treated at 45 °C.

Using a 1-mL disposable syringe and a thin catheter, 200 μL of each temperature-treated blood sample was introduced into a groove array via the cylinder house, which was connected to the inlet hole. The sample was allowed to flow through the cylinder house by applying a pressure difference of 20 cm H_2_O. Red cells passing through individual micro-channel arrays were monitored using an inverted metallographic microscope, a video-camera, and a video-recorder system. As shown in Figure 7a and b, microscopic images revealed good erythrocyte deformability in temperature-untreated samples subjected to MC-FAN. However, a marked decline in erythrocyte deformability was observed in blood samples treated at temperatures above 37 °C for 30 min. An example is shown in Figure 7c. Temperature-treated samples also demonstrated increased transit time for low transit sample volumes. Figure 8 illustrates some examples of blood fluidity of temperature-treated samples measured as transit time against transit sample volume. These graphs show that erythrocyte deformability decreased with increasing temperature over 37 °C, although there was considerable inter-individual variation.

**Figure 9.**
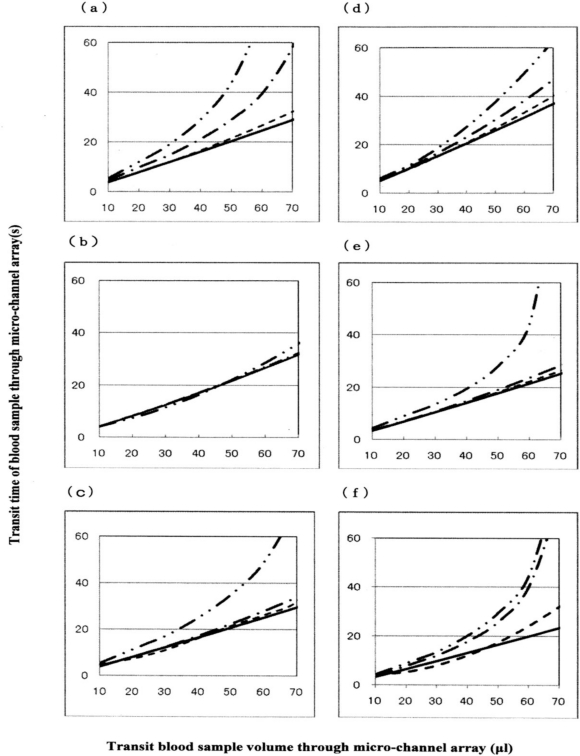
Blood fluidity in samples from six further donors measured in terms of transit time against transit sample volume. Solid lines represent the temperature-untreated control; broken lines represent samples treated at 37 °C; one-point lines represent samples treated at 42 °C; and two-point lines represent samples treated at 45 °C.

Blood was therefore obtained from further six healthy donors, and temperature-treated samples were compared with a temperature-untreated sample as a reference (Figure 9). The results were similar to those in Figure 8. Both sets of results (*n* = 10) are summarized in Table 1. The temperature-dependent decrease in erythrocyte deformability induced by mild heating was evaluated using Student’s *t*-test on the total sum of the difference in transit times between a given temperature-treated sample and each temperature-untreated reference, with varying transit sample volumes (20, 40 and 60 μL). Significant differences (*p* < 0.001) between the treated and control samples were evident for all samples treated at 45 °C with transit sample volumes of 20, 40 and 60 μL and all samples treated at 42 °C with transit sample volumes of 20, 40 and 60 μL. To cope with Figures 8 and 9, significant differences (*p* < 0.001) of the samples treated at 45°C (*i.e.*, two-point lines) and 42 °C (*i.e.*, one-point lines) against the control samples (*i.e.*, solid lines) were shown, respectively. We therefore concluded that erythrocyte deformability decreased with increasing temperature of the blood sample over 37 °C.

## 5. Discussion

Hemichrome is rarely found in erythrocytes *in situ*. However, spectroscopic analysis has shown that human HbO_2_ A from healthy donors tends to degrade to hemichrome, even at close to physiological temperatures and pH. However, this process is a function of pH, temperature and progress of autoxidation of ferrous HbO_2_ A to ferric metHb, through oxidation by bound oxygen. The findings suggest that autoxidation is inseparably related to the instability of Hb and its degradation to hemichrome.

As with autoxidation, Shikama [27–30] evaluated various mechanisms involving MbO_2_ and HbO_2_. Shikama clearly demonstrated that the autoxidation reaction does not simply involve the dissociative loss of O_2_^−^ from HbO_2_, but is rather caused by the nucleophilic displacement of O_2_^−^ from HbO_2_ by a water molecule or a hydroxyl ion that enters the heme pocket from the surrounding solvent. The iron is thus converted to the ferric met form, and the water molecule or hydroxyl ion remains bound to Fe (III) at the sixth coordinate position to form the aqua- or hydroxide-met species. A generalized pathway for this S_N_2 mechanism can be written using monomeric Mb (MbO_2_) as an example:

**Figure:**
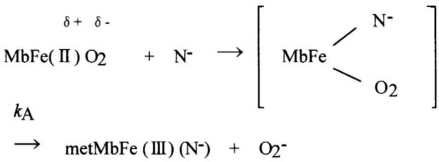

where *k*_A_ represents the rate constant of anion-induced autoxidation with nucleophilic anion displacement and N^−^ can be SCN^−^,F^−^,OCN^−^, N_3_^−^, or CN^−^, and *in vivo*, H_2_O or OH^−^. Here, anion-induced autoxidation with nucleophilic anion displacement of O_2_ results in an intermediate ferrous heme/anion complex that acts as an electron donor to displace oxygen.

In the framework of this S_N_2 mechanism and the accepted framework of hemichrome formation [9], hemichrome can form in physiologically normal Hb molecules by the following scheme:

**Figure:**
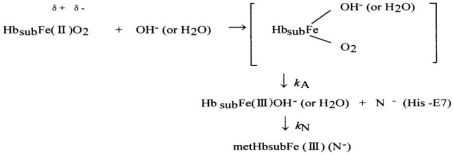

where Hb_sub_ represents each subunit of the Hb molecule. Nucleophilic displacement of O_2_^−^ by entry of a water molecule or a hydroxyl ion should be the rate-limiting step, and the subsequent conversion of the met form into hemichrome by a heme ligand (N^−^) endogenous to the protein must proceed very quickly with the kinetic relationship *k*_N_ >> *k*_A_. The most probable candidate for N^−^ in the HbA molecule is N^ɛ^-nitrogen of the distal His (E7) (the only amino acid side chain in the ligand pocket) of each subunit, because N^ɛ^-nitrogen is located more than 0.4 nm from the iron in Hb, and is therefore not expected to coordinate in native Hb [9].

It is significant to note that the content of metHb in normal red cells has been reported to be ≤1% [31,32]. An NADH-dependent enzyme system can reduce metHb to deoxy-ferrous Hb and prevent the continued accumulation of metHb resulting from oxidation of the bound O_2_ to the ferrous heme iron (II) [33–35]. This fact poses the question of how erythrocytes can elicit the range of responses to hemichrome with such a small amount of metHb. We suggest that this can be achieved by intramolecular anion-induced nucleophilic displacement of molecular dioxygen via an intermediate ferrous heme/anion complex (a low-spin hemichrome) [13]. Such a reaction would follow the scheme:

**Figure:**
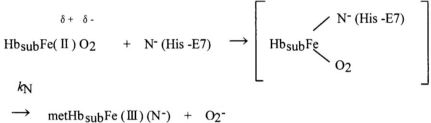

In this scheme, nucleophilic displacement can be caused within the heme pocket by N^ɛ^-nitrogen of the distal His (E7) instead of nucleophilic incursion of the water molecule or the hydroxyl ion from outside the molecule. As indicated earlier, hemichrome formation occurred at every stage during the autoxidation reaction of HbO_2_ A, *i.e.*, at the initial, intermediate, and final stages, as a function of the pH and temperature of the solution. While the reaction proceeds along this scheme, it is possible that hemichromes can be derived not only from HbO_2_ species, but also from deoxy Hb and metHb. Thus, vital hemichromes in erythrocytes *in situ* might arise from intramolecular anion-induced nucleophilic displacement of O_2_ via an intermediate ferrous heme/anion complex.

As aforementioned, Heinz bodies have been detected and characterized in drug-induced hemolytic anemia, defects in the intraerythrocytic reducing system (e.g., G-6-PD deficiency) and in unstable Hb disease [1,2]. Regarding the molecular pathogenesis of unstable Hb disease, the instability of labile Hb variants in patients can be attributed to amino acid substitutions (or deletions), which disrupt and perturb the Hb structure via interference with α-helix formation, disruption of heme binding, or altered α_1_β_1_ or α_2_β_2_ contacts [1]. The consequent changes in circulating red cells in patients include: an inherent tendency towards irreversible denaturation of Hb or globin due to a defect in the amino acid composition of the labile Hb molecules; a continuous tendency toward hemichrome formation; precipitation or aggregation of the molecules resulting in building up of the molecules to form Heinz bodies; and hemolysis.

Exposure of red cells to acetylphenylhydrazine and subsequent staining with crystal violet also revealed a greater abundance of Heinz bodies within G-6-PD deficient cells, compared with that in normal cells [36,37]. In addition, Sear *et al.* [38] and Campwala and Desforges [39] reported that Heinz bodies often appeared in normal aging red cells, and that this age-related appearance of Heinz bodies was especially pronounced in splenectomized individuals [40]. According to several authors [41–43], aged or damaged red cells affected by drugs may be filtered off by the spleen, irrespective of whether or not they contain Heinz bodies, in a similar manner to the filtering off of red blood cells in patients with unstable Hb caused by unstable Hb hemolytic anemia. The number of Heinz bodies formed in normal erythrocytes from freshly drawn venous blood from healthy donors increased with increasing temperature; when aliquots of venous blood were subjected to mild heating at temperatures over 37 °C for 30 min, Heinz bodies were visualized by exposing blood smears to acetylphenylhydrazine and staining with crystal violet.

The combination of *in situ* observations and experimental findings suggests that instability leading to hemichrome formation is not only a peculiarity of labile Hb variants, but is also an innate characteristic of physiologically normal Hb molecules. Hemichrome formation and subsequent Heinz body clustering can occur in normal red cells as a consequence of cell aging, oxidative stress, or the effects of destabilizing factors, such as pH and temperature. Amino acid substitutions (or deletions) characteristic of the Hb variants in unstable Hb disease [1], for instance, could reinforce the innate instability of normal Hb molecules.

**Figure 10.**
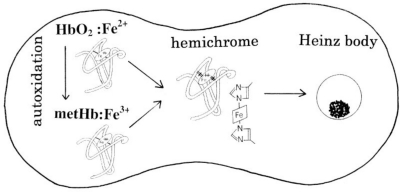
Diagram representing biosensing mechanisms in the spleen responsible for the removal of aged and damaged red cells from the blood circulation.

Figure 10 shows a diagram of the proposed biosensing mechanisms in the spleen that are responsible for the removal of aged and damaged red cells from the blood circulation. The suggested sequential changes occurring within normal red corpuscles upon the removal of non-functional erythrocytes include: a continuous tendency toward hemichrome formation; precipitation or aggregation of the molecules resulting in the formation of Heinz bodies; and hemolysis. To maintain senescent cell recognition or homeostasis in the blood circulation, Hb molecules might exert delicate control of the fate of erythrocytes via hemichrome formation and Heinz body clustering, through promotion of the innate molecular instability of physiologically normal Hb resulting in the production of oxidized or denatured Hb. The distal His (E7) at position 64 in the α chain and at position 94 in the β chain of the Hb molecule might play a key role in hemichrome formation. Heinz body-containing red cells become trapped in the spleen while traversing small apertures in the basement membranes separating the cords from the sinusoids [3], where the spaces are sufficiently small to require extreme deformation of red cells. We demonstrated that erythrocyte deformability decreased with increasing temperatures over 37 °C.

Normal erythrocytes only develop Heinz bodies late in their lifespan. A wide variety of biochemical changes have been reported to accompany red cell aging [44], including carboxymethylation of proteins, activation of proteases, glycosylation of proteins, loss of membrane area, decline in changes in the band 4.1a to 4.1b ratio, increases in oxidized lipids and proteins, changes in cell rheology and fragility, changes in exposure of cell surface sugars, and gradual accumulation of Ca^2+.^ With regard to this issue, Low [45] demonstrated that band 3 clustering might warrant closer scrutiny as a possible transducer of distress signals from the cytoplasm to the external surface of the cell, in light of the fact that it can be caused by hemichrome binding, ATP depletion, malondialdehyde formation, Ca^2+^ accumulation, oxidative cross-linking, or membrane skeletal weakening. It is known that hemichromes formed within erythrocytes bind to the cytoplasmic portion of band 3 in the membrane, then rapidly copolymerize with the soluble cytoplasmic domain of membrane band 3, forming an insoluble copolymer, followed by other changes involved in the pathogenesis of red cell destruction [46–50]. The dominant role of band 3 clustering suggests that hemichrome formation-induced band 3 clustering could also provide a key to the control of the fate of senescent and damaged red cells in the blood circulation.

The mechanisms shown in Figure 10 not only help to explain the fate of normal erythrocytes in the blood circulation, but also shed light on some clinical aspects of hemolytic anemia and its associated acute blood loss. The situation in patients with G-6-PD deficiency [1,2] can be used as an example. Red cells are known to be vulnerable to injury by endogenous and exogenous oxidants. Oxidants can be inactivated by reduced glutathione in erythrocytes with normal G-6-PD activity, because the pentose phosphate shunt supplies NADPH, which is required for glutathione recycling. In G-6-PD deficient erythrocytes, however, the reduced glutathione cannot be restored and the cells sustain irreversible oxidative damage. However, G-6-PD deficiency only produces symptoms when the patient is exposed to an environmental factor that results in increased oxidative stress, including drugs such as antimalarials (e.g., primaquine, pamaquine, dapsone), sulfonamides, nitrofurantoin and phenacetin. The resulting crisis can lead to hemolysis of up to 25–30% of the red cells within hours. However, the crisis is self-limited, and only the older population of red blood cells is destroyed. The mechanisms responsible for such an acute hemolytic crisis in patients taking these drugs cannot be explained on the basis of conventional views. However, Figure 10 suggests that this crisis could be triggered by fluctuations in endogenous and exogenous oxidative stress under the fragile pentose phosphate shunt.

Malaria provides another example. This is a protozoal disease transmitted by the bite of female *Anopheles* mosquitoes (*Plasmodium falciparum*, *P. malariae, P. vivax, and P. ovale*). Blackwater fever syndrome is caused by infection with *P. falciparum*, and is characterized by repeated bouts of chills and fevers, severe intravascular hemolysis and anemia, jaundice, hemoglobinuria (black urine) and splenic enlargement [51–53]. Patients often develop fevers above 40 °C. A feature of this disease is that the symptoms can be exacerbated in defected patients with G-6-PD deficiency as a result of taking quinine. In these patients, massive destruction of the red cells infected by the parasites occurs, but similar numbers of normal erythrocytes are also ruptured, resulting in characteristic hemoglobinuria. The reason for the massive hemolysis of non-infected red cells is unclear. However, the possibility of a relationship between quinine ingestion and the associated massive hemolysis, and the relevance of G-6-PD deficiency are addressed in Figure 10. Fever above 40°C, an endogenous G-6-PD deficiency, and exogenous quinine ingestion all represent conditions that induce acute hemolysis, thus leading to massive blood loss and hemoglobinuria.

## 6. Conclusions

(1) This overview has discussed the molecular biosensing mechanisms in the spleen, where hemichrome formation and subsequent Heinz body clustering within erythrocytes play key roles in the removal of aged and damaged red cells from the blood circulation.

(2) Spectrophotometric evidence suggests that the autoxidation of Hb molecules was inseparably related to the instability of Hb and its degradation to hemichrome.

(3) Based on the accepted S_N_2 mechanism for autoxidation of human HbO_2_ A and the accepted framework for hemichrome formation, we concluded that vital hemichromes in erythrocytes might arise from intramolecular anion-induced nucleophilic displacement of O_2_ via an intermediate ferrous heme/anion complex in physiologically normal Hb molecules.

(4) In this reaction scheme, the most probable candidate for such an intramolecular anion in normal Hb molecules is the N^ɛ^-nitrogen of the distal His (E7), since N^ɛ^-nitrogen is located more than 0.4 nm from the iron.

(5) Accordingly, nucleophilic displacement could be caused within the heme pocket by the N^ɛ^-nitrogen of the distal His (E7) of the Hb molecule, instead of nucleophilic incursion of a water molecule or hydroxyl ion from outside the molecule.

(6) Thus, the N^ɛ^-nitrogen of the distal His (E7) at position 64 of the α chain and position 94 of the β chain can delicately control the fate of red blood corpuscles through the removal of non-functional erythrocytes from the circulation by causing hemichrome formation and subsequent Heinz body clustering. The rigid intraerythrocytic hemichrome inclusions act as “sticking points” in the spleen, and hence Heinz body-containing red cells become trapped and undergo hemolysis.

## Figures and Tables

**Table 1.** Summary of the mean ± standard deviation of transit time of blood samples (s) for a given transit sample volume (μL). Prior to application of MC-FAN, blood samples were subjected to mild heating at 37 °C, 42 °C and 45 °C for 30 min, respectively. The number of subjects was 10. Significant differences (*p* < 0.001) of the temperature-treated samples against the temperature-untreated reference were shown as **.

**The mean ± standard deviation of transit time of blood samples (s) for a given transit sample volume (μl)**			
	Transit blood sample volume (μL)		
	20 μL	40 μL	60 μL
Temperature-untreated reference	8.0 ± 1.08	16.3 ± 2.24	25.1 ± 3.50
Temperature-treated at 37°C	7.8 ± 1.35	16.4 ± 2.57	26.4 ± 3.42
Temperature-treated at 42°C	8.5 ± 1.38**	18.3 ± 2.94**	36.3 ± 6.32**
Temperature-treated at 45°C	9.3 ± 2.46**	21.2 ± 5.99**	41.8 ± 13.23**

## Abbreviations

G-6-PD: glucose 6-phosphate dehydrogenase;
Hb: hemoglobin;
Mb: myoglobin;
NADH: nicotinamide adenine dinucleotide, reduced form;
MES: 2-(*N*-morpholino) ethanesulfonic acid monohydrate.
